# Efficient Prodrug Activator Gene Therapy by Retroviral Replicating Vectors Prolongs Survival in an Immune-Competent Intracerebral Glioma Model

**DOI:** 10.3390/ijms21041433

**Published:** 2020-02-20

**Authors:** Shih-Han Chen, Jui-Ming Sun, Bing-Mao Chen, Sheng-Che Lin, Hao-Fang Chang, Sara Collins, Deching Chang, Shu-Fen Wu, Yin-Che Lu, Weijun Wang, Thomas C. Chen, Noriyuki Kasahara, Hsin-Ell Wang, Chien-Kuo Tai

**Affiliations:** 1 Section of Neurosurgery, Department of Surgery, Ditmanson Medical Foundation Chia-Yi Christian Hospital, Chia-Yi 600, Taiwan; shihhan63@yahoo.com.tw (S.-H.C.); 07178@cych.org.tw (J.-M.S.); 2 Department of Biotechnology, Asia University, Taichung 413, Taiwan; 3 Department of Biomedical Sciences, National Chung Cheng University, Chia-Yi 621, Taiwan; ben731209@yahoo.com.tw (B.-M.C.); lin.regadam@gmail.com (S.-C.L.); hfc3a01@gmail.com (H.-F.C.); biodcc@ccu.edu.tw (D.C.); biosfw@ccu.edu.tw (S.-F.W.); 4 Department of Neurological Surgery, University of California, San Francisco, CA 94143, USA; Sara.Collins2@ucsf.edu (S.C.); Noriyuki.Kasahara@ucsf.edu (N.K.); 5 Department of Health and Nutrition, Chia Nan University of Pharmacy and Science, Tainan 717, Taiwan; 04688@cych.org.tw; 6 Department of Neurosurgery, University of Southern California, Los Angeles, CA 90033, USA; weijunwa@usc.edu (W.W.); tchen68670@gmail.com (T.C.C.); 7 Department of Radiation Oncology, University of California, San Francisco, CA 94143, USA; 8 Department of Biomedical Imaging and Radiological Sciences, National Yang-Ming University, Taipei 112, Taiwan

**Keywords:** brain tumor, retroviral replicating vector, prodrug activator, gene therapy, *E. coli* nitroreductase gene

## Abstract

Prodrug activator gene therapy mediated by murine leukemia virus (MLV)-based retroviral replicating vectors (RRV) was previously shown to be highly effective in killing glioma cells both in culture and in vivo. To avoid receptor interference and enable dual vector co-infection with MLV-RRV, we have developed another RRV based on gibbon ape leukemia virus (GALV) that also shows robust replicative spread in a wide variety of tumor cells. We evaluated the potential of GALV-based RRV as a cancer therapeutic agent by incorporating yeast cytosine deaminase (CD) and *E. coli* nitroreductase (NTR) prodrug activator genes into the vector. The expression of CD and NTR genes from GALV-RRV achieved highly efficient delivery of these prodrug activator genes to RG-2 glioma cells, resulting in enhanced cytotoxicity after administering their respective prodrugs 5-fluorocytosine and CB1954 in vitro. In an immune-competent intracerebral RG-2 glioma model, GALV-mediated CD and NTR gene therapy both significantly suppressed tumor growth with CB1954 administration after a single injection of vector supernatant. However, NTR showed greater potency than CD, with control animals receiving GALV-NTR vector alone (i.e., without CB1954 prodrug) showing extensive tumor growth with a median survival time of 17.5 days, while animals receiving GALV-NTR and CB1954 showed significantly prolonged survival with a median survival time of 30 days. In conclusion, GALV-RRV enabled high-efficiency gene transfer and persistent expression of NTR, resulting in efficient cell killing, suppression of tumor growth, and prolonged survival upon CB1954 administration. This validates the use of therapeutic strategies employing this prodrug activator gene to arm GALV-RRV, and opens the door to the possibility of future combination gene therapy with CD-armed MLV-RRV, as the latter vector is currently being evaluated in clinical trials.

## 1. Introduction

Glioblastoma multiforme (GBM) is the most frequent form of primary malignant brain tumor in adults [1], and it tends to aggressively invade the surrounding brain tissue so as to make complete surgical resection virtually impossible. Clinical trials of GBM gene therapies using a conventional replication-defective retroviral vector encoding Herpes simplex virus thymidine kinase with subsequent administration of the prodrug ganciclovir did not achieve any improvement in patient survival [2], which was attributed to extremely low levels of tumor transduction. Replication-competent viral vectors enable enhanced tumor transduction levels, since these viral vectors can replicate and multiply after the initial infection event, and each infected tumor cell in effect becomes a viral vector producer cell [3,4,5,6,7,8]. We and others have previously shown that amphotropic murine leukemia virus (MLV)-based retroviral replicating vectors (RRV) achieve highly efficient and tumor-selective gene transfer to glioma cells both in culture and in vivo [9,10,11]. Furthermore, intracerebral or intravenous injection of MLV-RRV resulted in little or no detectable infection in the normal brain or extracerebral tissues [12,13,14]. Early phase clinical studies of RRV-mediated prodrug activator gene therapy have shown highly promising results in recurrent high-grade glioma patients [15,16], and an international Phase III trial is currently on-going.

Highly efficient and tumor-selective gene transfer achieved by RRV enables efficacious prodrug activator gene therapy, which involves expression of enzymes that can convert precursor prodrugs into active chemotherapeutic drugs within the transduced tumor cells. This essentially forces the tumor to self-generate the chemotherapy drug from within. However, due to the relatively restricted payload capacity of RRV, which can only accommodate approximately 1.3-kb of additional transgene sequence inserted into the full-length replication-competent viral genome, it has generally been difficult to insert more than a single therapeutic gene per vector. For prodrug activator gene therapy, this is the equivalent of single-agent chemotherapy generated directly within the tumor.

Analogously, since combination chemotherapy with multiple drugs is generally more efficacious than single-agent chemotherapy, there may be potential to achieve greater efficacy if multiple RRV encoding different prodrug activator genes could be combined. However, in this case the retrovirus envelope sequence in different vectors may need to be varied, so that these vectors do not compete for the same cell surface receptor, a phenomenon which in retrovirology is classically described as “receptor interference”. In particular, MLV-RRV currently in clinical development expresses the MLV 4070A strain amphotropic envelope, which binds the ubiquitous inorganic phosphate transporter, PiT-2 [17]. In contrast, gibbon ape leukemia virus (GALV) envelope enables cellular entry through another member of the same phosphate transporter family, PiT-1, and accordingly we have developed GALV-based RRV which also appears to show robust replicative spread in a wide variety of cancer cell lines [18,19,20,21,22].

To evaluate the potential of GALV-RRV as a therapeutic agent for glioma therapy, we incorporated the yeast cytosine deaminase (CD) [23] or *E. coli* nitroreductase (NTR) [24,25] prodrug activator genes into this type of vector. The CD enzyme converts 5-fluorocytosine (5-FC) to the highly toxic metabolite 5-fluorouracil [26], which is a potent antineoplastic agent routinely used in cancer therapy. The NTR enzyme converts 5-(aziridin-1-yl)-2,4-dinitrobenzamide (CB1954) to a 4-hydroxylamino derivative that is subsequently converted into a potent cytotoxic bifunctional alkylating agent [27].

To explore the use of GALV-RRV for prodrug activator gene therapy of glioma, in these studies we used an immune-competent rodent model of intracerebral glioma to examine tumor growth as well as survival after intracerebral inoculation of GALV-RRV carrying CD and NTR genes, followed by treatment with their respective prodrugs. We evaluated whether the high transduction level and persistent gene expression achieved by GALV-RRV has the potential to enhance the efficacy of prodrug activator gene therapy.

## 2. Results

### 2.1. GALV-RRV Achieves Highly Efficient Transgene Delivery to Glioma Cells

**Figure 1:**
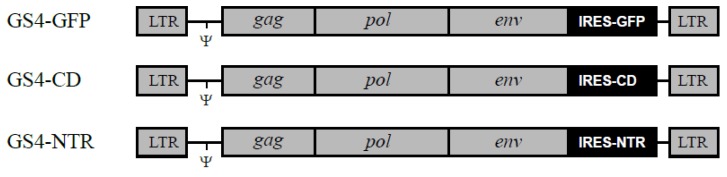
Structure of gibbon ape leukemia virus (GALV)-retroviral replicating vectors (RRV) carrying transgenes. Each GALV-RRV contains an internal ribosome entry site (IRES)-green fluorescent protein (GFP), IRES-cytosine deaminase (CD) or IRES-*E. coli* nitroreductase (NTR) gene expression cassette inserted between GALV *env* and the 3′ untranslated region (UTR). Ψ, packaging signal. LTR, long terminal repeat.

**Figure 2:**
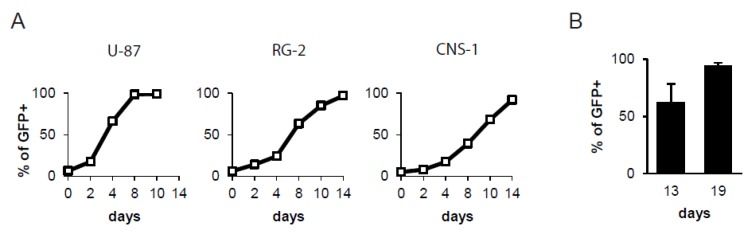
Replicative spread of GS4-GFP in glioma cells in culture and in intracerebral gliomas in vivo. (**A**) GS4-GFP-transduced RG-2 cells (5%) and uninfected RG-2 cells (95%) were mixed and seeded onto culture plates. At various time points after cell mixing, the cell populations were analyzed for GFP expression. X-axis: days after cell mixture. Y-axis: % of cells expressing GFP. (**B**) GS4-GFP (2 × 10^4^ TU) was injected into a pre-established intracerebral RG-2 tumor model in Fischer 344 rats. The spread of GS4-GFP in tumors was examined by quantification of GFP expression at 13 (*n* = 6) and 19 days (*n* = 3) after viral vector inoculation.

As previously described, the GS4-GFP vector contains a wild-type GALV provirus into which an internal ribosome entry site (IRES)-green fluorescent protein (GFP) gene expression cassette has been inserted precisely between GALV *env* and the 3′ untranslated region (UTR) [28] (Figure 1). To examine the ability of GS4-GFP to replicate in glioma cells in culture, we infected human (U-87) and rat (RG-2, CNS-1) glioma cells with the viral vector at low multiplicities of infection (MOI) and confirmed by flow-cytometric analysis that GS4-GFP could transduce each glioma cell line. We further studied the replication kinetics of GS4-GFP in each glioma cell line by initially mixing 5% infected glioma cells with 95% uninfected glioma cells, and examining horizontal transmission of GFP expression over time. Flow-cytometric analysis showed that GS4-GFP could efficiently transduce each glioma cell line, and spread throughout the entire cell population within 8 days in the case of human U-87 cells, and within 14 days in the case of rat RG-2 and CNS-1 cells (Figure 2A).

### 2.2. Efficient and Progressive Spread of GALV-RRV in an Intracerebral Glioma Model

We evaluated the ability of GALV-based RRV to achieve a high level of transduction in gliomas in vivo by testing the GS4-GFP vector in an intracerebral RG-2 tumor model. A single dose of 2 × 10^4^ transducing units (TU) of GS4-GFP was injected into pre-established intracerebral RG-2 tumors in syngeneic Fischer 344 rats. At two time points after vector injection, intratumoral spread of GS4-GFP was evaluated by flow-cytometric analysis of excised intracerebral tumors. On day 13 post-vector injection, the percentage of GFP-positive RG-2 tumor cells averaged 62.0% ± 16.4%, and by day 19 post-vector injection, this had increased to 94.2% ± 2.8% (*p* < 0.01), demonstrating that GS4-GFP spread efficiently and progressively in orthotopic intracerebral rat gliomas (Figure 2B).

### 2.3. No Detectable Spread of GALV-RRV to Extratumoral Tissues

To detect any possible GALV-RRV spread to extratumoral tissues, real-time PCR assay of genomic DNA extracted from various extratumoral organs was performed using primers specific for the GALV *env* (Table 1). This assay could detect down to 50 copies of the GALV provirus per 5×10^4^ cell genomes, representing a transduction level of approximately 0.1%. As expected, GALV sequence could be readily detected in GS4-GFP-transduced glioma tissues (*n* = 3; 19 days after viral vector injection). However, no detectable spread was observed in any of the extratumoral organs examined from the same GS4-GFP-injected animals.

### 2.4. Dose-Dependent Cytotoxicity of RRV-Transduced Cells after Prodrug Administration

**Figure 3:**
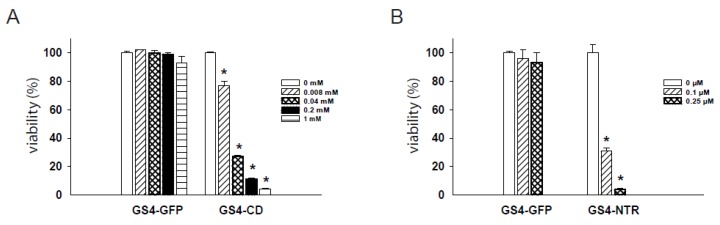
In vitro cytotoxicity achieved by GALV-based RRV plus prodrug treatment. (**A**) GS4-CD- and GS4-GFP-transduced RG-2 cells were exposed to 5-FC ranging from 0 to 1 mM, and cell viability was determined 3 days later by MTS assay. (**B**) GS4-NTR- and GS4-GFP-transduced RG-2 cells were exposed to CB1954 ranging from 0 to 0.25 μM, and cell viability was determined 3 days later by MTS assay. *, *p* < 0.005.

To examine the ability of RRV to deliver prodrug activator genes, GS4-CD- and GS4-NTR-transduced RG-2 cells were first treated with their respective prodrugs, 5-FC and CB1954, at various concentrations. To distinguish the cytotoxic effects of prodrug activator gene function from any potential nonspecific toxicity due to the prodrugs themselves, GS4-GFP-transduced cells were also treated with each prodrug at the corresponding concentrations as experimental controls. The 5-FC and CB1954 prodrugs showed negligible cytotoxicity at concentrations of ≤1 mM and ≤0.25 μM, respectively. Infection with GS4-CD or GS4-NTR resulted in potent killing of RG-2 cells exposed to the corresponding prodrug at all concentrations tested, and the degree of cytotoxicity was highly prodrug concentration-dependent (Figure 3). Exposing GS4-CD-transduced cells to 5-FC at 0.04 mM led to a dramatic drop in cell viability to ~30% relative to the control group. Similarly, exposing GS4-NTR-transduced cells to CB1954 at 0.1 μM induced the same degree of cytotoxicity, indicating that the NTR/CB1954 enzyme prodrug system might also be useful if delivered in the context of RRV to inhibit tumors and prolong survival.

### 2.5. RRV-Mediated Prodrug Activator Gene Delivery Significantly Improves Survival of Immune-Competent Rats Bearing Gliomas

**Figure 4:**
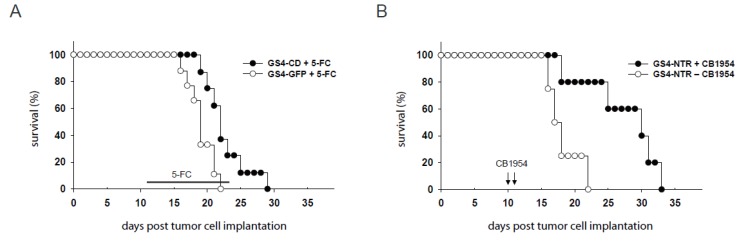
Survival analysis of immune-competent rats bearing intracerebral RG-2 gliomas. (**A**) GS4-CD or GS4-GFP was stereotactically injected into pre-established intracerebral RG-2 tumors three days after tumor inoculation. Eight days after viral vector inoculation, the rats received intraperitoneal injections of 5-FC (100 mg/kg), once every other day, for a total of 7 treatments. Survival curves were constructed for two treatment groups: GS4-CD plus 5-FC, and GS4-GFP plus 5-FC. (**B**) GS4-NTR was stereotactically injected into pre-established intracerebral RG-2 tumors three days after tumor inoculation. Seven and eight days after viral vector inoculation, the rats received daily intraperitoneal injections of CB1954 (2.5 mg/kg) or PBS. Survival curves were constructed for two treatment groups GS4-NTR plus CB1954 and GS4-NTR plus PBS.

We determined whether the high in vivo tumor transduction levels achieved by GALV-RRV has the potential to improve survival by evaluating prodrug activator gene therapy using GS4-CD and GS4-NTR in the intracerebral RG-2 glioma model. After establishing intracerebral RG-2 gliomas by stereotactic implantation, the GS4-CD or GS4-NTR vector was inoculated via intratumoral injection, as above. Treatment with the GS4-CD vector, followed by a single cycle of 5-FC administered by intraperitoneal injection, resulted in a survival advantage with a median survival time of 22 days, as compared to a median survival time of 19 days in the control group treated with the GS4-GFP vector and 5-FC (*p* < 0.05) (Figure 4A). Treatment with the GS4-NTR vector followed by two doses of CB1954 administration also resulted in a significant survival advantage, with a median survival time of 30 days, compared to the median survival time of 17.5 days in the control group (*p* < 0.05) (Figure 4B).

### 2.6. MicroPET Imaging of RG-2 Glioma-Bearing Rats after Prodrug Activator Gene Therapy

**Figure 5:**
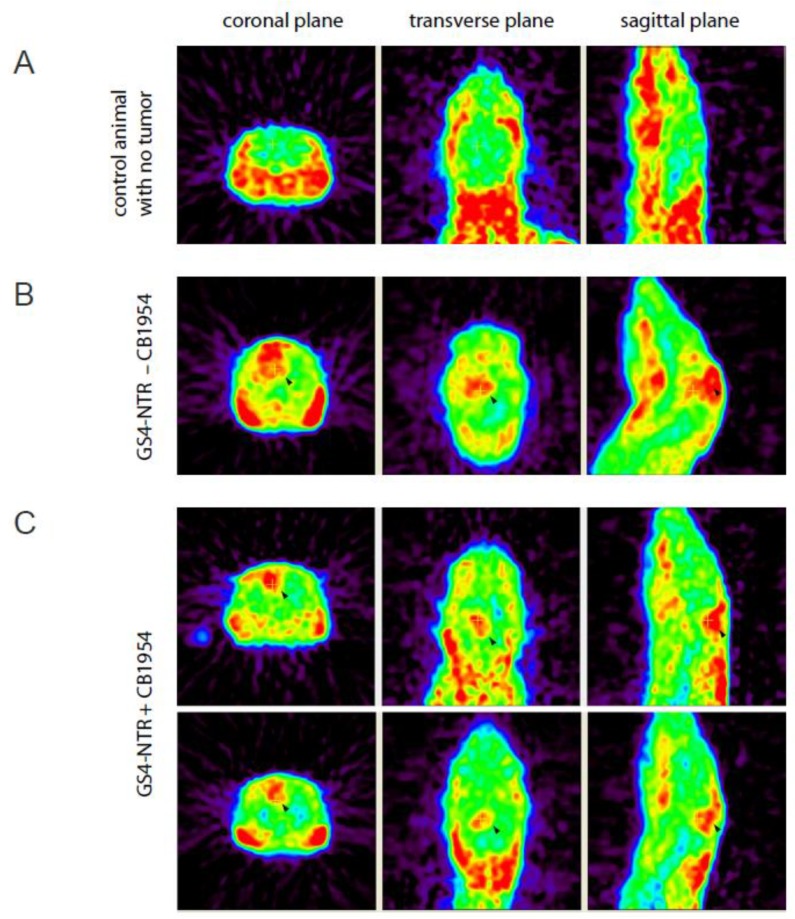
MicroPET imaging of intracerebral RG-2 glioma-bearing rats. MicroPET imaging of L-[^18^F] FET in rats was performed using the R4 system, 20 days after intracerebral RG-2 glioma implantation. Representative examples comparing microPET imaging results from non-tumor-bearing rats (**A**), tumor-bearing rats treated with GS4-NTR but without prodrug administration (**B**), or tumor-bearing rats with GS4-NTR and CB1954 prodrug treatments (**C**) are shown, and tumor regions are indicated by arrowheads. The tumor uptake of L-[^18^F] FET in the GS4-NTR−CB1954 group relative to the GS4-NTR + CB1954 group was 2.015-fold (*p* < 0.01).

Our previous study reported that *O*-2-[^18^F] fluoroethyl-L-tyrosine (L-[^18^F] FET) microPET was superior to [^18^F] FDG microPET for the monitoring of brain tumor due to the low uptake of L-[^18^F] FET in normal brain [29]. MicroPET imaging of non-tumor-bearing (Figure 5A) and glioma-bearing (Figure 5B,C) rats using L-[^18^F] FET revealed sizable RG-2 gliomas at the tumor implantation site in the group treated with GS4-NTR alone. A clear difference between tumor-implanted and normal brains was observed. In contrast, rats treated with GS4-NTR plus CB1954 showed smaller tumors remaining at the tumor implantation site, demonstrating the therapeutic benefit achieved by GALV-based RRV-mediated prodrug activator gene therapy.

## 3. Discussion

Persistent nonlytic infection of tumor cells by RRV facilitates the widespread seeding of prodrug activator genes, thereby allowing synchronized cell killing triggered by prodrug administration. Using GALV-based RRV expressing the CD and NTR suicide genes followed by the administration of the prodrugs 5-FC and CB1954, we have achieved highly efficient killing of glioma cells both in culture and in vivo, resulting in significantly prolonged survival in an immune-competent intracerebral glioma model.

In addition to the significant survival improvement achieved by GALV-based RRV-mediated CD prodrug activation therapy, here we also demonstrate a significant tumor inhibitory effect on intracerebral RG-2 tumors after GS4-NTR-mediated prodrug activation therapy. Of note, the *E. coli* NTR isoform B (NfsB) gene cloned into the GS4-NTR vector expresses an enzyme that actively converts CB1954 to a 4-hydroxylamino derivative that is subsequently converted into a potent cytotoxic bifunctional alkylating agent capable of cross-linking DNA and therefore achieving cell-cycle-independent killing of both actively proliferating and non-proliferating cells [30]. 

However, despite significant increases in survival, all animals did succumb to tumor burden. Factors which may have contributed to the lack of eradication of tumor burden and potential avenues for improvement include the vector dose and/or the schedule of prodrug delivery. In vivo vector replication and spread was assessed with approximately 60% of tumor cells positive for GFP expression (vector) 13 days post vector administration. In this study the animals were treated with prodrug beginning day 7 (NTR) or day 8 (CD), where the number of tumor cells transduced would be less than 60%. Increasing the vector dose and hence the number of cells transduced at the time of prodrug administration may increase the survival benefit.

Here we have validated both CD and NTR as effective prodrug activator genes in the context of GALV-RRV. As noted, the different cytocidal effects caused by CD/5-FC and NTR/CB1954 would allow us to employ combination prodrug activation therapy, analogous to combination chemotherapy, but generated directly within the tumor itself, thereby avoiding adverse effects of systemic chemotherapy. However, for optimal combined gene therapy with both prodrug activator genes, these genes must be delivered using RRV with different envelopes in order to avoid receptor interference. Since MLV-RRV and GALV-RRV utilize different cellular receptors (PiT-2 and PiT-1 phosphate transporters, respectively) for viral entry [17], co-infection of glioma cells with both vectors supplied with different prodrug activator genes may be employed to achieve synergistic cytotoxic effects, thus augmenting the efficacy of gene therapy [18,22]. Now that the present studies have validated the use of GALV-RRV expressing NTR as a single-agent prodrug activator vector in itself, in future studies we can proceed to evaluate combination prodrug activator gene therapy together with the current clinical vector, CD-armed MLV-RRV.

Real-time PCR analysis demonstrated that the replication of GALV-RRV is highly restricted to the tumor itself, with no spread to ectopic sites such as bone marrow and spleen detectable in immune-competent glioma models. This result is consistent with our previous study showing that MLV-RRV delivered to intracerebral RG-2 tumors in immune-competent syngeneic hosts showed no detectable spread to all normal tissues examined [13]. Our inability to detect RRV in extratumoral tissues suggests that, although impaired innate and adaptive immunity in cancer cells enable progressive replicative spread of the virus within tumors even in an immune-competent host [31,32], these mechanisms remain intact in normal tissues and prevent systemic RRV dissemination. Of course, it is possible that low levels of systemic RRV dissemination might occur below the detection limit of our PCR assay, and the potential for retroviral vectors to cause insertional mutagenesis that can contribute to the development of malignancies remains a concern [33,34,35]. However, when considering the use of RRV as an agent for cancer therapy, this concern is alleviated, as incorporation of prodrug activator genes not only arms the vector against cancer cells, but also helps to eliminate inadvertently transduced normal cells that might become transformed. Furthermore, as an additional safety mechanism, various antiretroviral drugs such as 3’-azido-3’-deoxythymidine (AZT) could be used to effectively block retroviral replication and dissemination. It should be noted however that, to date, more than 300 patients with recurrent high-grade glioma have been treated with CD-armed MLV-RRV in multiple Phase I dose escalation trials (NCT01156584, NCT01470794, NCT01985256) and an international Phase III trial (NCT02414165), and there have been no such severe adverse effects related to malignant transformation. Certainly, clinical development of GALV-RRV will require further confirmation of preclinical safety, biodistribution and therapeutic efficacy. Nonetheless, given the extremely poor prognosis of patients with GBM, the use of RRV may represent a promising treatment strategy, particularly if the therapeutic benefits outweigh the potential risks, as indicated by our current results.

## 4. Materials and Methods

### 4.1. Viral Vectors and Cell Lines

As described previously, plasmid pGS4-GFP [28] encodes a replication-competent GALV vector, in which an IRES-GFP gene cassette has been inserted between GALV *env* and the 3′ UTR. The CD and NTR genes were amplified from plasmid pACE-CD [12] and *E. coli* genomic DNA by PCR, and used to replace the GFP sequence in pGS4-GFP, generating plasmids pGS4-CD and pGS4-NTR, respectively. The primer sequences used for PCR amplification of the *E. coli* NTR gene (isoform B) are 5′-atggatatcatttctgtcgcct-3′ and 5′-ttacacttcggttaaggtgatgtt-3′ [36]. The transformed human embryonic kidney cell line 293T [37], U-87 human glioma cells (obtained from the American Type Culture Collection), and RG-2 and CNS-1 rat glioma cells were grown in Dulbecco’s modified Eagle’s medium (Invitrogen, Carlsbad, CA, USA) supplemented with 10% fetal bovine serum (Invitrogen). Viral vectors were produced by transient transfection of 293T cells with plasmid pGS4-GFP, pGS4-CD or pGS4-NTR using Lipofectamine 2000 Reagent (Invitrogen). For in vitro transduction experiments, 4 μg/mL polybrene (Sigma, St. Louis, MO, USA) was added to the culture medium at the time of infection. Titer determination was performed on target cells in the presence of AZT (Sigma) to prevent secondary vector replication, as described previously [38]. Virus titers were represented as transducing units (TU)/mL.

### 4.2. Viral Vector Replication Assays in Glioma Cells

Glioma cells at 20%–25% confluency in six-well plates were grown in fresh medium containing GS4-GFP virus stock at low MOI. At various time points post-infection, the cells were analyzed for GFP expression by flow cytometry. This procedure was performed to ensure that the entire cell population exhibited GFP fluorescence. Infection of GS4-CD and GS4-NTR to RG-2 cells was performed in parallel until full transduction was achieved. In a separate experiment, GS4-GFP-transduced glioma cells were mixed with uninfected glioma cells at a proportion of 5% of the total cell population and seeded onto six-well plates. At various time points post-infection, the cell populations were analyzed for GFP expression.

### 4.3. Viral Vector Replication Assay in Intracerebral Glioma Model

All animal studies were performed according to institutional guidelines under protocol #1030413 (25 December 2014) approved by the Institutional Animal Care and Use Committee at National Chung Cheng University. Intracerebral RG-2 gliomas were established by stereotactic injection of 5 × 10^4^ RG-2 cells into the right frontal lobe in Fischer 344 rats (National Laboratory Animal Center, Taipei, Taiwan) as described previously [13]. Three days later, the rats were stereotactically injected with 2 × 10^4^ TU of GS4-GFP at the tumor implantation site. At various time points after viral vector transduction, the rats were sacrificed and the tumors were excised and digested with collagenase (Invitrogen). The dissociated cells were filtered through 100-μm cell strainers, pelleted by centrifugation, resuspended in culture medium containing 50 μM AZT, and plated onto culture plates. After overnight culture, the cells were trypsinized and immediately subjected to flow cytometry for GFP expression analysis.

### 4.4. Real-Time PCR Analysis

To detect any integrated GALV-RRV sequence in tissue genomes, real-time PCR was performed as described previously [19,39] using an ABI Prism 7700 sequence detector. The primers 5′-cctattactcctccttctgttg-3′ and 5′-gggcctgatatttttgtctaag-3′ were designed to target GALV *env*. Apolipoprotein B gene for precise amounts of input genomic DNA were also quantified as an internal control (primers: 5′-cacgtgggctccagcatt-3′ and 5′-tcaccagtcatttctgcctttg-3′). Real-time PCR was done in 25 µL of reaction mixture containing genomic DNA, 12.5 μL of 2× SYBR green real-time PCR master mix (Toyobo, Osaka, Japan) and 300 nM of each primer. Products were amplified by 35 cycles of successive incubation at 95 °C for 15 s and at 60 °C for 1 min. A standard curve for GS4-GFP copy number was generated by amplification of serially diluted GS4-GFP plasmid template at specific copy numbers mixed into genomic DNA from uninfected rat cells.

### 4.5. In Vitro Cytotoxicity Assay

GS4-GFP-, GS4-CD- and GS4-NTR-transduced RG-2 cells were seeded onto replicate 96-well plates (2000 cells/well), cultured overnight, and exposed to 5-FC (Sigma) or CB1954 (Sigma) at various concentrations. Cell viability was determined 3 days later by MTS assay using the CellTiter Aqueous One Solution Cell Proliferation Assay kit (Promega, Madison, WI, USA).

### 4.6. Survival Assay Using Intracerebral Glioma Models

The GS4-CD and GS4-NTR viral vectors (2 × 10^4^ TU) were injected 3 days after intracerebral tumor implantation (5 × 10^4^ RG-2 cells) in Fischer 344 rats. Eight days after GS4-CD vector injection, intraperitoneal injections of 5-FC (100 mg/kg) were performed, once every other day, for a total of 7 treatments. For the GS4-NTR group, CB1954 (2.5 mg/kg) was injected intraperitoneally 7 and 8 days after vector injection.

### 4.7. MicroPET Imaging

L-[^18^F] FET microPET imaging of tumor-bearing rats was performed 20 days after RG-2 implantation using the R4 system (Concorde Microsystems, Knoxville, TN, USA) as described previously [29]. Static images were acquired 60 min after the intravenous injection of 3.7 MBq of L-[^18^F] FET in the rats.

### 4.8. Statistical Analysis

Student’s *t*-test was used for statistical analysis of in vitro cytotoxicity results. Kaplan-Meier analysis and log-rank tests were used to evaluate survival. All analyses were conducted using SAS software (SAS Institute, Cary, NC, USA).

## Figures and Tables

**Table 1:** Biodistribution of GS4-GFP after intratumoral injection of the vector in immune-competent intracerebral glioma model.

	Copies of GALV *env* Per Cell
tumor	1.46 ± 0.23
contralateral normal brain	−
bone marrow	−
spleen	−
intestine	−
liver	−

Genomic DNA extracted from intracerebral glioma and extratumoral organs of GS4-GFP-infected rats was analyzed by real-time PCR assay. The numbers of GALV *env* copies per cell are presented as means ± standard deviations. −, not detectable (detection limit was 0.001 copy per cell).
